# Automatic Segmentation of Intraluminal Thrombus in Abdominal Aortic Aneurysms Based on CT Images: A Comprehensive Review of Deep Learning-Based Methods

**DOI:** 10.3390/jcm14238497

**Published:** 2025-11-30

**Authors:** Jia Guo, Fabien Lareyre, Sébastien Goffart, Andrea Chierici, Hervé Delingette, Juliette Raffort

**Affiliations:** 1 Clinical Chemistry Laboratory, University Hospital of Nice, 06107 Nice, France; jia.guo@inria.fr (J.G.); sebastien.goffart@inria.fr (S.G.); raffort-lareyre.j@chu-nice.fr (J.R.); 2 Epione Team, INRIA, Université Côte d’Azur, Sophia Antipolis, 06902 Nice, France; herve.delingette@inria.fr; 3 Department of Vascular Surgery, Hospital of Antibes, 06600 Antibes, France; 4 Laboratory of Molecular Physio Medicine (LP2M), UMR 7370, CNRS, University Côte d’Azur, 06107 Nice, France; chierici.ap@chu-nice.fr; 5 Digestive Surgery Unit, Archet 2 Hospital, University Hospital of Nice, 06200 Nice, France

**Keywords:** deep learning, abdominal aortic aneurysm, intraluminal thrombus, imaging segmentation, CTA, EVAR

## Abstract

**Objectives**: This review aims to review the application of deep learning (DL) techniques in the imaging analysis of abdominal aortic aneurysm (AAA), with a specific focus on the segmentation of intraluminal thrombus (ILT). **Methods**: A comprehensive literature review was conducted through searches of PUBMED and Web of Science up to September 2025. Only English-language studies applying DL-based networks for ILT segmentation in patients with AAA on computed tomography angiography were included. After screening 664 articles, 22 met the eligibility criteria and were included. The reported methodological frameworks and segmentation performance metrics were extracted for comparison and analysis. **Results**: Among the studies included, the reported Dice similarity coefficients ranged from 0.81 to 0.93 for 2D networks and from 0.804 to 0.9868 for 3D networks. Notably, 2D Multiview fusion models outperform other 2D approaches, while 3D U-Net remains a strong baseline. Methods using preoperative images demonstrated great applicability for surgical planning, while postoperative segmentation faced challenges related to imaging artifacts caused by stent. **Conclusions**: This review provides a comprehensive overview of recent DL-based ILT segmentation methods for AAA patients on CTA, offering perspectives for applications in advanced preoperative planning and postoperative surveillance. Despite the promising results, the lack of standardized datasets limits model development and external validation. Future research should address these limitations by focusing on multicenter standardized datasets and seamless integration into clinical workflows.

## 1. Introduction

Abdominal aortic aneurysm (AAA) is a permanent, localized dilation of the abdominal aorta, characterized by a transverse diameter exceeding 3 cm or a more than 50% increase compared to normal size [1,2,3,4]. Most AAAs are fusiform and contain thrombi [5]. While typically asymptomatic, rupture remains the most feared complication, with mortality rates exceeding 80% [6,7]. Risk increases with aneurysm diameter, necessitating timely diagnosis and treatment [7]. Definitive treatment involves surgical repair via open aneurysm repair (OAR) or endovascular aneurysm repair (EVAR) [8], which demands precise pre-surgical planning. AAA imaging is important in both diagnosis and management. Duplex ultrasound (DUS) is the standard imaging method for screening and surveillance [3,9,10], while computed tomography angiography (CTA) is preferred for preoperative planning due to its high resolution, ability to provide 3D images, excellent contrast, and rapid acquisition [9,11,12].

Aortic segmentation serves two primary purposes as follows: preoperative support by accurate diagnosis and surgical planning and postoperative monitoring for prognosis assessment. It enables morphological observation and facilitates indirect measurement, valuable for evaluating aortic dimensions and the related conditions. Therefore, the accurate segmentation of both the aorta and intraluminal thrombus (ILT) is essential for comprehensive morphological assessment for informed preoperative planning as well as follow-up post-intervention. The segmentation of ILT is an important step as it can affect volume calculation and contribute to the evaluation of wall stress modeling and EVAR feasibility assessment. Traditionally, segmentation has been performed manually, which is time-consuming, labor-intensive, and subject to intra- and inter-observer variability [13]. These challenges highlight the need for automated, robust, and reproducible methods.

ILT, a complex aggregate of blood, platelets, and cellular debris, is a major component of AAA. These elements, involved in blood coagulation, are usually in free circulation within vessels, but several phenomena can lead to aggregation and thrombus formation, such as tissue damage or hemodynamic disturbances [14]. ILT contributes to the progression of AAAs [15], and its growth is strongly associated with aneurysm expansion and increased rupture risk [16]. In addition, the location and extent of ILT in AAA help define the proximal and distal landing zone during EVAR and are taken into consideration when determining where the stent can be securely anchored. Thus, ILT serves as an indirect anatomical factor affecting both EVAR feasibility and long-term sealing efficacy [10]. Long-term postoperative surveillance is necessary after EVAR to detect failures or complications early [17,18]. Aortic and ILT remodeling is analyzed to detect postoperative complications such as endoleaks or graft migration. According to EVAR reporting standards [19], an increase in aneurysm diameter or volume of 5% or more is usually considered a clinical failure after EVAR. An increase in the volume of ILT can potentially contribute to AAA sac growth. On the contrary, in successful cases, ILT within isolated regions may continue to shrink and eventually disappear. Therefore, the segmentation and analysis of ILT directly support patient-specific EVAR planning, risk stratification, and surveillance, impacting clinical decision-making.

On CTA, the ILT is characteristically visualized as a hypoattenuating, non-enhancing region relative to the contrast-filled lumen, reflecting the absence of contrast agent penetration. It has received relatively limited attention compared to lumen segmentation or aneurysm sac segmentation. Traditional segmentation approaches, including semi-automatic tools like ITK-SNAP [20], are available for aortic segmentation, but they require considerable manual effort to accurately delineate ILT, which limits their efficiency and applicability in clinical settings. Artificial intelligence (AI)-based techniques show considerable promise in overcoming these limitations. The application of AI in vascular diseases, including AAA, has gained significant momentum in recent years. Previous reviews [21] have highlighted AI’s potential to automate key processes such as image segmentation, aneurysm detection, and risk stratification. Over the past decade, research has evolved from traditional semi-automated techniques [22,23] to sophisticated deep neural networks, particularly U-Net variants and hybrid architectures tailored to vascular structures. A variety of deep learning models are increasingly being employed for aortic segmentation in medical images, overcoming the shortcomings of manual and semi-automatic segmentation methods [21,24]. These models have demonstrated superior performance in aortic segmentation tasks, further addressing the challenges associated with manual annotations. Beyond improving efficiency, deep learning (DL)-based approaches can reduce inter-observer variability and facilitate large-scale, standardized analyses of vascular images. Multi-structure segmentation of the vessels refers to the simultaneous delineation of multiple anatomical or pathological components within the vascular region, allowing us to analyze distinct substructures, including the lumen and vessel wall, as well as thrombus and calcifications.

This review summarizes current DL applications for the segmentation of ILT in CT images of AAA patients. By narrowing the scope to this multi-structure segmentation task, distinct from general vascular segmentation, the review aims to synthesize and compare existing DL architectures, report their performances, analyze methodological aspects that influence model generalizability, and explore the clinical readiness and translational potential of these approaches. Through this integrative perspective, this review provides a comprehensive understanding of the current progress, persisting challenges, and future directions for meaningful clinical AI-based vascular segmentation.

## 2. Methods

### 2.1. Search Strategy

A literature search was conducted through PubMed and Web of Science according to the PRISMA (Preferred Reporting Items for Systematic Reviews and Meta-Analyses) guidelines. These two databases provide extensive coverage of both biomedical and interdisciplinary research relevant to the present topic, and they include the majority of journals indexed in broader platforms. PubMed provides high-quality biomedical coverage, while Web of Science captures engineering and computer science research. Although specialized technical databases such as IEEE Xplore or the ACM Digital Library were not searched, preliminary checks confirmed that key AI-related publications from IEEE were already indexed in Web of Science, supporting the adequacy of this database selection strategy.

J.G. and A.C. reviewed articles published from January 2016 to September 2025 using a combination of the following terms: “artificial intelligence”, “deep learning”, “neural network”, “segmentation”, “measurement”, “aneurysm”, “aortic aneurysm”, “abdominal aortic aneurysm”, and “intraluminal thrombus” (Supplementary Table S1). The starting point of January 2016 was chosen because bibliometric analyses have demonstrated that the majority of publications on these topics have emerged within the past 5 to 8 years, reflecting the rapid evolution and growing focus on artificial intelligence and its applications in this field during this period [25,26]. In case of any disagreement concerning article inclusion, a third reviewer (J.R.) was in charge of the final decision.

### 2.2. Eligibility Criteria

Article inclusion criteria were established following the population (P), intervention (I), comparison (C), and outcome (O) approach [27]. The inclusion criteria were original articles written in English including participants diagnosed with AAA in which a DL-based method was applied for aortic segmentation, including individual ILT segmentation based on CT/CTA imaging data. Non-peer reviewed studies, reviews, comments, or pre-prints were excluded. Studies were included if they involved ILT segmentation, regardless of whether they also performed segmentation of other vascular structures such as the aortic lumen or vessel wall. The inclusion criteria also required that at least one performance metric was reported in the study to allow analysis and comparison of results. Within this framework, the “comparison (C)” component referred to the reference standard used for evaluating model performance, typically manual expert annotation or, in some cases, conventional segmentation methods. The “outcome (O)” was defined as quantitative segmentation performance metrics. These outcomes were extracted to enable consistent evaluation and comparison. A checklist for eligibility criteria was developed to assess the eligibility of each study (Supplementary Table S2). The list and definition of the performance metrics is also reported in Supplementary Table S3.

### 2.3. Data Collection Process

Data extraction was performed using a standardized extraction in an Excel sheet. Data extraction from the included articles was primarily conducted by one reviewer (J.G.) and independently verified by a second reviewer (A.C.). Extracted variables included study characteristics (publication year, imaging modality, and dataset information), methodological details (segmentation target, network architecture, and validation strategy), and performance metrics. The initial data extraction was conducted by one reviewer and independently verified by a second reviewer. Any inconsistencies were resolved through discussion and consensus. The authors of the original studies were not contacted for missing data or the clarification of performance metrics.

### 2.4. Synthesis Methods

Given the heterogeneity in study designs and outcome reporting, a comprehensive synthesis was performed. Studies were categorized according to network dimensionality (2D versus 3D architectures) and clinical context (preoperative versus postoperative imaging). Within each category, we summarized the segmentation performance metrics and compared the results across different learning or training strategies. In addition, we considered methodological aspects including model training, approaches to addressing data scarcity, and validation strategies. The synthesis further highlights how these AI-based segmentation methods may be integrated into clinical workflows and their potential applicability in real-world settings.

## 3. Results

**Figure 1:**
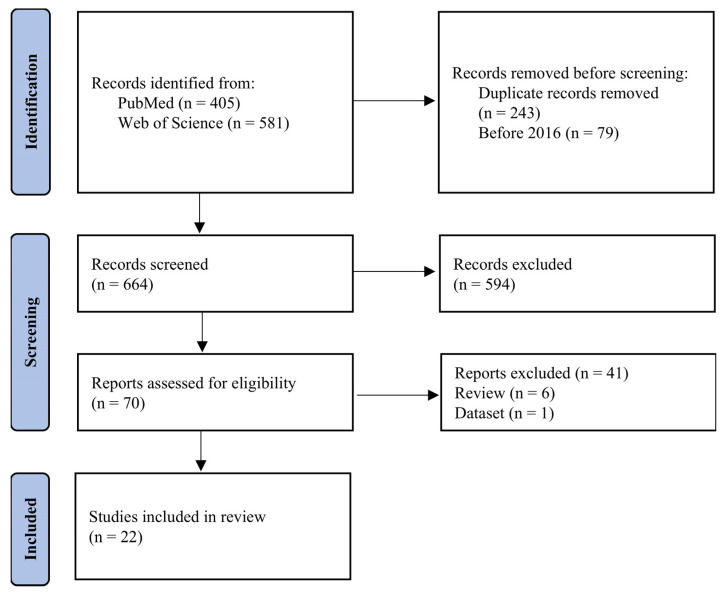
Flowchart indicating the process for the literature search and selection of the studies for intraluminal thrombus (ILT) segmentation on CT/CTA images from patients with abdominal aortic aneurysm (from 2016 to 2025).

The research identified 405 articles from PubMed and 581 articles from Web of Science. After screening the titles and abstracts, 70 articles were selected for full-text evaluation. Following the application of predefined eligibility criteria, 22 articles were deemed eligible for inclusion. A flowchart summarizing the search and selection process is presented in Figure 1, and the results are shown in Table 1.

### 3.1. 2D Deep Learning Network

Early-stage medical image segmentation often employed 2D approaches [28,29,30,32,33,34,35,36,37,39,40,42,43,45] due to their reduced computational cost and lower demand on GPU resources. The 2D model processes each axial slice independently, which simplifies training and inference. However, a major limitation of 2D segmentation lies in its inability to leverage inter-slice spatial continuity—an essential component for accurately capturing anatomical structures with complex three-dimensional morphology. As a result, 2D models are prone to producing discontinuous or anatomically inconsistent segmentations across adjacent slices. To bridge the performance gap between 2D and 3D methods while managing computational efficiency, several different strategies have been integrated. These can be broadly categorized into the following: (1) 3D construction based on 2D DL network segmentation [28,30], (2) multimodality fusion [29], (3) integrating deep learning models with traditional image processing or shape-constrained priors [32,33,36], (4) combining results or features from multiple-view 2D slices [34,45] to form a “2.5D” approach, and (5) explicitly designing networks or training regimes to extract latent inter-slice spatial features [35]. Such strategies aim to combine the computational efficiency of 2D models with the spatial awareness characteristic of 3D architectures. The following section summarizes representative methods in each category.

To improve the consistency across the 2D slices’ segmentation results, Lopez-Linares et al. [28] proposed a 2D fully convolutional network (FCN) for the automatic detection and segmentation of thrombus based on 13 post-EVAR CTA scans. A 3D reconstruction algorithm for conventional image processing was then applied with the 2D probability maps for the 3D mask generation, achieving a DSC of 0.82 ± 0.07. It demonstrated strong robustness to metal artifacts and offered rapid inference (35 slices/s), supporting its potential for clinical deployment in post-surgical monitoring.

In parallel, to explore more information from the patients, Wang et al. [29] proposed a 2D dual U-Net fusion model that integrates features from multimodality to segment the aorta wall, lumen, thrombus, and calcium deposits in 22 CT and magnetic resonance imaging (MRI) pairs. The model that fuses the encoder features not only improves training speed and allows for the shared representation of multimodality images to be learnt but also shows a slight improvement in comparison with the model with a single modality. Then, a method that integrated deep learning models with traditional image processing or shape-constrained priors [32,33] could also improve the segmentation performance. This research also showed that neurons identifying AAA structures are modality-independent, resulting in improved accuracy over single-modality networks. This means that although we may not be able to collect a lot of CT data for DL training, the application of multimodal data can effectively improve network performance.

Moreover, several studies have combined DL with traditional priors or expert systems to enhance segmentation robustness. Lareyre et al. [33] proposed a hybrid method combining a feature-based expert system [55] and convolutional neural network (CNN)-based refinement for fully automated segmentation of the abdominal vascular system. Based on 40 contrast-enhanced and 53 lower-contrast CTAs of infrarenal AAA patients, it achieved high accuracy, DSC = 0.89 for the ILT on 623 slices, with improved performance compared to the expert system. Caradu et al. [32] proposed a two-stage pipeline of U-Net to segmentate the lumen and the ILT separately. The ILT segmentation, realized based on the lumen boundary as a prior, achieved a DSC of 0.81 ± 0.10 with a dataset of 100 preoperative CTAs (13465 slices) from patients with infrarenal AAAs. This research also resulted in a commercial software, PRAEVAorta, which is classified as a Class IIb medical device and received U.S. FDA 510(k) clearance as “Automated Radiological Image Processing Software.” Alternatively, in the multi-stage pipeline that employs a 2D Residual U-Net with dilated convolutions proposed by Abdolmanafi et al. [36], segmentations were refined using prior segmentation to reduce background interference. With the segmentation of the aortic wall, lumen, ILT, and calcification, the method was trained with 6030 CT slices from 56 AAA patients and additionally evaluated with 19 new patients, resulting in classification accuracies >98% for all components. Segmenting on masked images that exclude surrounding organs with similar intensities enhances the network’s focus on aortic sub-elements.

In a separate study, to capture spatial consistency without full 3D modeling, Brutti et al. [34] proposed a multi-view integration approach. This method was evaluated on 85 pre-operative CTA scans with AAA as primary pathology and achieved a DSC of 0.89 ± 0.04. It was based on a previous method [56], which integrated three separate 2D models for aortic segmentation in slices of different views, including axial, sagittal, and coronal views. The same team [45] later extended the geometric analysis, aiming at screening AAA. The combined approach mitigates challenges related to poorly defined boundaries and similar-intensity neighboring structures, effectively resulting in a 2.5D segmentation framework.

Additionally, to benefit the spatial features between the slices, Jung et al. [35] proposed a Bi-Directional Convolutional Long Short-Term Memory (Bi-CLSTM)-based ITL segmentation method leveraging volumetric coherence in postoperative CTA images. Validated on a large-scale AAA dataset of 60 patients, the method demonstrated superior performance (DSC of 0.89) over both 2D and 3D CNN approaches. By integrating spatial features via Mask R-CNN [57] and sequential context through Bi-CLSTM, it effectively addressed challenges from artifacts, noise, and ambiguous boundaries, while reducing false negatives, a key factor in clinical robustness. In their ablation study, Mask R-CNN achieved the highest performance (DSC = 0.8636, Jaccard index = 0.7666) among the evaluated models, including 2D U-Net, 2D U-Net++, and mHED, showing an improvement of at least 0.03. Compared to the baseline Mask R-CNN without Bi-CLSTM, the incorporation of Bi-CLSTM led to notable improvements as follows: the DSC increased from 0.8636 to 0.8730, the Jaccard index from 0.7666 to 0.7809, and the false negative rate reduced from 0.1238 to 0.1069.

A large-scale 2D model has also been developed for the segmentation of ILT and aortic lumen. Wang et al. [39] integrated a DeepLabv3+-based 2D DCNN model with a ResNet-50 backbone based on preoperative CTA scans from 340 AAA patients. Benefiting from transfer learning from non-medical domains such as ImageNet, the model achieved a mean intersection over union (IOU) of 0.9078 and ILT IOU of 0.8650, with volumetric differences from manual segmentation below 5%. These studies utilized substantially larger datasets compared to previous works, highlighting the generalizability and robustness of the proposed approach.

Overall, 14 studies employed 2D segmentation methods for ILT delineation, reporting DSCs ranging from 0.81 to 0.93. Among these approaches, the pipeline integrating U-Net-based localization with a 2.5D CNN and multi-view feature integration [45] achieved the highest performance with a DSC of 0.93.

### 3.2. 3D Deep Learning Network

In contrast to 2D approaches, 3D segmentation models operate directly on volumetric data, enabling the extraction of spatial features across all three dimensions. This capability allows the network to better capture anatomical context, reduce boundary inconsistencies between slices, and improve the fidelity of structure delineation. Building on these advantages, several 3D segmentation networks have been developed for AAA analysis [41,44,46,47,48,54]. While the baseline models of 3D U-Net offer strong performance [41,46,48], cascaded architectures [44], Multiview integration [47], and dynamic linking mechanisms [54] have further improved segmentation accuracy and robustness.

Kongrat et al. [41] implemented a segmentation approach based on a standard 3D U-Net [58] architecture to reconstruct both lumen and thrombus regions from CTA data. The model was trained on 60 annotated CTA volumes, including normal cases, AAA with or without thrombus, and using extensive data augmentation including geometric and grayscale transformations. The model achieved high segmentation accuracy, with a DSC of 0.9868 on the testing set. Experimental results demonstrated that the 3D U-Net effectively delineates both large and small thrombus regions, highlighting its potential as a reliable and efficient tool for 3D AAA screening and assessment.

Building upon the U-Net architecture, Mu et al. [44] proposed a fully automatic multi-class segmentation approach for AAA from CTA using a novel Context-Aware Cascaded U-Net (CACU-Net) based on 70 CTA scans. The two-stage architecture combines a Residual 3D U-Net and an auto-context 3D U-Net, enhanced by dilated convolutions, an anisotropic context module, and hierarchical supervision to capture both short- and long-range dependencies, achieving DSCs of 0.945 and 0.804 for lumen and ILT, respectively. The resulting morphological metrics closely matched those from expert segmentation, underscoring its potential as a reliable and fully automated tool for clinical AAA assessment. Based on previous research [44,59], Lyu et al. [47] proposed an automated thrombus segmentation workflow using a novel mixed-scale-driven Multiview perception network (M2Net), which integrates axial, coronal, and sagittal views’ segmentation to improve the recognition of low-contrast thrombus regions. Training with a dataset of 80 CTA scans, the model achieved a DSC of 0.884 for ILT segmentation. The network architecture incorporates both the original CTA volume and the three views’ 2D segmentation outputs as inputs, enriching the spatial and contextual information available to the second-stage model. Functionally, the network serves as an integration mechanism that fuses multi-view segmentations to produce a more robust and accurate final output.

Independently, Zhang et al. [54] proposed SDLU-Net, a novel similarity-based dynamic linking network designed for the automated segmentation of AAA and the associated branching vessels based on 63 CTA scans. The architecture integrates a dynamic linking mechanism that adaptively establishes long-range dependencies based on feature similarity, addressing challenges such as irregular aneurysm morphology and vessel bifurcation continuity. Evaluated on a dataset of 13 CTA scans, including complex cases with branching and aneurysmal deformation, the model achieved a DSC of 0.828 for ILT. SDLU-Net effectively captures the spatial features across slices by sampling 3D patches and training them using a 3D convolutional network. While not explicitly designed for inter-slice feature extraction, this sampling and modeling strategy implicitly achieves that goal.

Compared with the 2D method, only six have reported the use of 3D approaches, with DSCs ranging from 0.804 to 0.9868. The original 3D U-Net combined with data augmentation was implanted in various studies and demonstrated good performance.

### 3.3. Preoperative and Postoperative Image

**Figure 2:**
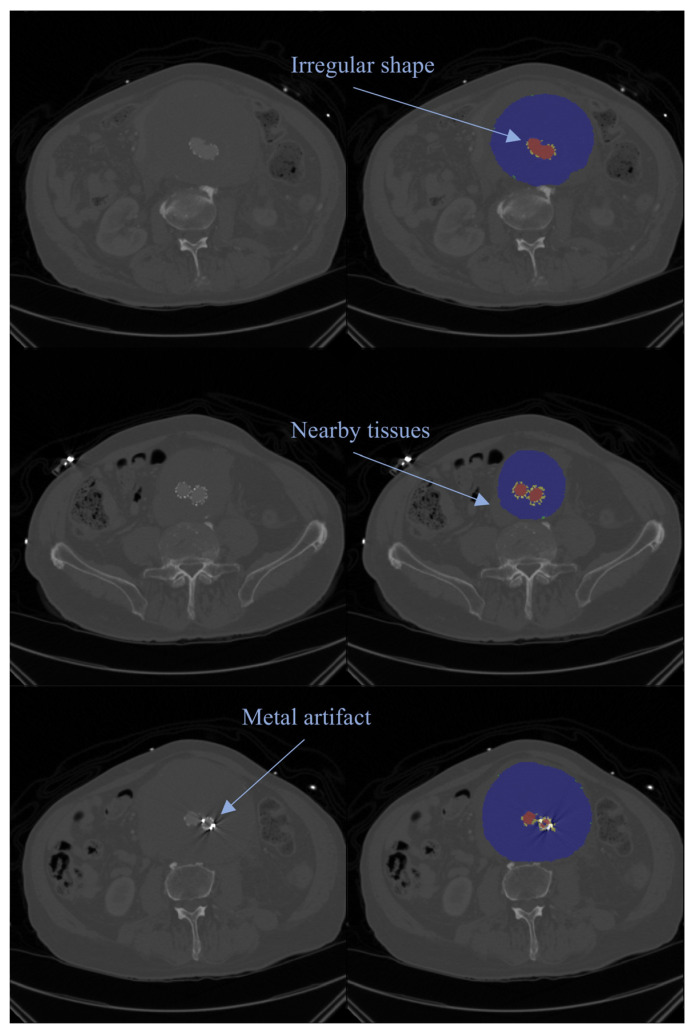
Representative postoperative CTA images showing axial slices. Left column: original CTA images; right column: corresponding manual segmentations (red: lumen; blue: ILT; yellow: stent grafts). The arrows highlight major challenges encountered in ILT delineation, including irregular thrombus morphology, interference from nearby tissues, and artifacts caused by metallic implants, all of which can compromise segmentation accuracy.

Depending on the clinical context, existing segmentation approaches can be broadly categorized into two types as follows: preoperative and postoperative segmentation [28,30,31,35,37]. These two scenarios involve distinct imaging characteristics and technical challenges. Preoperative scans generally present clearer vascular anatomy with minimal artifacts, offering a more stable imaging environment. Postoperative CTA scans frequently contain metallic artifacts caused by stents graft, as well as altered vascular morphology resulting from EVAR, both of which significantly complicate the segmentation process [30,37], as shown in Figure 2. On CTA, the luminal surface of the ILT often presents irregular morphology and complex boundaries. Following EVAR, the metallic stent graft not only renders the luminal surface of the ILT less smooth but also introduces substantial artifacts. Imaging artifacts typically do not affect the high-attenuation lumen; however, the low-attenuation ILT is more susceptible to artifact distortions, often appearing as conspicuous dark regions in the image. These artifacts arise from several physical and technical limitations of CT imaging, outlined as follows: high-attenuation metallic structures induce photon starvation and beam hardening, resulting in prominent streak artifacts; partial volume effects and the limited point spread function of the scanner cause the artificial thickening of stent struts, known as blooming artifacts; and reconstruction inaccuracies and scatter radiation contribute to the shape distortion of the vascular lumen and reduced visibility of adjacent structures [60,61]. Collectively, these artifacts degrade image quality, further obscuring the already ill-defined margins of the ILT and considerably complicating its detection and precise segmentation.

To overcome these challenges, recent studies have explored advanced DL-based segmentation architectures incorporating Mask R-CNN [35,37] and multi-scale feature extraction [28,30,31,37]. These approaches are designed to better capture the ILT region and enhance the robustness and accuracy of segmentation on postoperative imaging, ultimately supporting improved clinical decision-making in AAA management.

The two-stage framework proposed by Lopez-Linares et al. [28] has shown effectiveness in segmenting post-EVAR scans. The first stage performs a coarse detection of the aneurysm region, including the ILT, while the second stage refines the segmentation by leveraging local contrast enhancement, enabling the more precise delineation of thrombus boundaries. In their follow-up study [30], they demonstrated that training separate models for pre- and postoperative scans led to improved segmentation accuracy, underscoring the value of tailored training strategies in AAA follow-up. In a related but distinct approach, Hwang et al. [37] proposed a DL framework for thrombus detection and segmentation based on an improved Mask R-CNN. Mask R-CNN uses a multi-task learning framework, where a shared backbone network extracts multi-scale features for parallel detection and segmentation tasks. This design promotes synergy between the tasks as follows: segmentation provides a more fine-grained pixel-wise supervision while detection offers spatial constraints. The features shared between tasks contribute to building a more robust and generalized model. By integrating a novel bounding box regression loss and a modified focal loss, their method achieved superior detection (F1 Score of 0.9197) and segmentation results (DSC of 0.8267) on a dataset comprising 60 postoperative CTAs. Building on this work, the same team later proposed the Bi-CLSTM-based method [35]. Although PRAEVAorta software [32] was developed based on preoperative data, it has been widely validated using morphological measurement across various clinical settings with postoperative data as follows: 101 CT scans within 48 postoperative CTs [38], in follow-up CTAs of 49 patients who underwent EVAR for infrarenal AAA [62], in 56 patients who underwent operation for imaging follow-up [63], in 762 multicenter CTA images of 499 postoperative of patients with a juxtarenal or infrarenal AAA [64], and 60 CTA and non-contrast computed tomography (NCCT) pairs comprising diverse postoperative subjects [65].

### 3.4. Data Lack in ILT Segmentation

The scarcity of high-quality annotated datasets for DL training is a huge challenge in AAA segmentation. Most existing studies rely on relatively small and institution-specific cohorts, which may not adequately reflect the full spectrum of anatomical variability encountered in AAA presentations across diverse populations. Several public abdominal datasets, such as AVT [52], the Vascular Model Repository [51], TotalSegmentator [49], and challenge AortaSeg24 [66], provide AAA annotations, but these are limited to lumen segmentation without ILT or calcification. A detailed comparison of these datasets is summarized in Table 2. This limitation arises because the manual segmentation of ILT is extremely time-consuming, and its low CT values of ILT, which is similar to those of adjacent AAA tissues, reduce the effectiveness of semi-automatic segmentation methods. The following section reviews representative approaches that mitigate data scarcity and annotation burdens, including synthetic data generation via generative models [31,43], active learning (AL) strategies [46], and distributed training on multiple datasets [48].

To solve this question, several studies [31,43] proposed the use of the generative method to enrich the dataset for the DL training. Lopez-Linares et al. [31] proposed a synthetic shape model generation using realistic deformations for data augmentation. This augmentation was used in the train of a 3D CNN [67], extended from prior research [28], for 28 infrarenal AAA segmentations. The synthetic shape model enables the generation of a wide variety of CTA scans for training. Using less than half of the original CTA scans, the 3D CNN trained with synthetic data accurately segments real aneurysms, achieving comparable DSCs and Jaccard coefficients to those obtained with real scans. To address the limitations of contrast-enhanced imaging in elderly populations, Chandrashekar et al. [43] employed Cycle-GAN and Conditional-GAN models to synthesize CTA from NCCT. Based on the previous study [42], the resulting system enabled accurate measurements of aortic diameter, lumen area, and thrombus volume, demonstrating strong agreement with manual assessment and highlighting a path toward contrast-free AAA evaluation and advancing a potential contrast-free pathway for AAA evaluation.

The long annotation time of AAA CTA is also a limitation when working with large datasets. Kim et al. [46] developed an AL-based DL pipeline for the automatic segmentation of AAA components, including the aortic lumen, thrombus, calcification, and vessels, based on a single site dataset of 300 preoperative CTA AAA scans. In the AL procedures, AL-corrected segmentation reduced the segmentation time by 13.74 ± 2.16 min compared to manual segmentation. However, this study did not compare the network performance between cases with and without Al, limiting the quantitative understanding of its relative benefit, except for the time saved.

While collecting public datasets from multiple sources can help mitigate data scarcity, challenges remain due to inconsistencies in annotation quality and the limited availability of large-scale datasets tailored to specific anatomical regions or disease types. Robbi et al. [48] developed an automated CTA analysis pipeline, named Blood Vessels Recognition and Aneurysms Visualization Enhancement (BRAVE), that makes strategic use of multiple public datasets [49,50,51,52,53] at different stages of its workflow. Each stage was trained on a distinct dataset [49,50] and forms a complete automatic procedure including two different DL network and three conventional methods, allowing the system to better handle variations in image quality, annotation style, and scanning protocols. The method permits vascular system segmentation by separating it into 19 anatomical regions, showing good performances (ILT DSC of 0.97 ± 0.03) on 20 full-abdomen pre-CTA scans from AAA patients collected from publicly available datasets [49,51,52,53], demonstrating its adaptability across heterogeneous data environments.

Despite recent advances in synthetic data generation and active learning techniques, the scarcity of high-quality, well-annotated ILT datasets continues to pose a major limitation for model development and validation. Limited sample diversity may introduce sampling bias and hinder the model’s ability to generalize across imaging protocols, scanners, and patient populations. Furthermore, training on small or homogeneous datasets increases the risk of overfitting, where models achieve high internal accuracy but fail to maintain performance in external settings. Addressing these challenges will require multicenter collaborations, harmonized annotation protocols, and open-access data initiatives to ensure reproducibility and enhance the clinical reliability of DL-based AAA segmentation.

## 4. Discussion

Recent AI-based segmentation methods have shown promise for ILT delineation, with the added capability of simultaneously segmenting other AAA components, including the aortic lumen and calcification. This advancement facilitates comprehensive morphological analysis and supports the automated extraction of key anatomical metrics, where early work [32,42,43,44,45] primarily focused on basic morphological metrics such as aneurysm length, maximum diameter, and volume. Kim et al. [46] further extended this by identifying anatomical landmarks for stent graft selection. Robbi et al. [48] performed a comprehensive morphological analysis that extracted anatomical metrics useful for EVAR planning, including the main requirements outlined in the 2024 ESVS guidelines [9]. Segmenting ILT and calcifications from preoperative scans enables clinicians to gain a more detailed understanding of the aneurysmal morphology, supporting more informed preoperative planning and accurate endograft sizing. Wall shear stress (WSS) is influenced by the shape and composition of ILT [68], and it could be estimated through ILT segmentation [69]. Changes in ILT morphology and thickness are not only structural observations, but they also reflect the physiological state of the aneurysm wall, especially the inflammatory activity and tissue degradation [70,71]. Furthermore, integrating postoperative segmentation results with clinical and biological data enables the control of postoperative changes and the development of more personalized and risk-adapted follow-up strategies. An increased thrombus load is associated with a higher likelihood of rapid expansion [72]. However, in the follow-up of EVAR, low thrombus burden is associated with increased rates of persistent type II endoleak, especially in the presence of a patent inferior mesenteric artery [73]. Thrombus density progressively increased in all patients after EVAR, more markedly in those with endoleak, showing a positive association with aneurysm volume in the presence of endoleak but a negative association in its absence [74].

For real world clinical applications, the external validation of AI models is required to ensure robustness and generalizability. However, not all studies have fully considered this requirement. Robbi et al. [48] evaluated their pipeline with 20 AAA pre-CTA scans from four publicly available datasets [49,51,52,53], three of which were not used during the entire training [51,52,53]. The implementation of DL networks as software tools greatly facilitated access for external users, enabling broader external validation. In particular, the commercial development of PRAEVAorta [32] has greatly expanded its external validation across various clinical settings and independent cohorts [38,62,63,64,65]. The software of the model enables the method to be widely applied and tested with different datasets. These consistent findings support the scalability and clinical readiness of commercialized segmentation software. In addition to technical validation, the implementation of such applications in clinical practice requires adherence to rigorous regulatory frameworks, including approval or clearance by authorities such as the U.S. Food and Drug Administration (FDA) and the European CE marking process [75]. These regulatory pathways ensure that AI-based medical devices meet established standards of safety, efficacy, and data transparency before clinical deployment. Successful clinical translation also depends on adequate training and acceptance by radiologists and vascular specialists [76]. Educational programs and hands-on workshops would help clinicians understand model outputs, interpret uncertainty, and identify potential limitations.

DL based segmentation for ILT in AAA varies considerably in both design and clinical applicability. Two-dimensional networks remain widely adopted due to their computational efficiency and flexibility, especially with huge datasets. However, their inability to capture inter-slice continuity limits performance in anatomically complex regions. Therefore, Multiview fusion approaches achieved the highest performance among 2D methods [45]. Other strategies, including hybrid approaches, 2.5D modeling, or sequential networks such as Bi-LSTM, etc., strike a balance by capturing spatial context without full volumetric processing. In contrast, 3D architectures provide improved spatial consistency, but they often require extensive labeled data and higher computational resources, making them less practical for institutions with a limited infrastructure. Among these, the original 3D U-Net remains a cornerstone approach because of its strong baseline performance and well-established architecture, making it an almost unavoidable choice in segmentation tasks. Additionally, while multimodality fusion has shown potential in integrating complementary anatomical information, its clinical deployment is restricted by the limited availability of multimodal imaging in routine clinical workflows.

Despite these advances, several limitations remain that hinder the widespread clinical adoption and methodological robustness of DL segmentation. Most existing studies rely on relatively small, single-center datasets acquired using institution-specific imaging protocols, which limits the generalizability of the trained models. The heterogeneity among public datasets, such as variations in annotation standards, segmentation targets, and anatomical coverage, further complicates model development and limits the utilization of the multicenter dataset for training. Most CT volumes are acquired with 1–5 mm, despite scanners being capable of providing higher-quality images. Moreover, a critical limitation is the lack of external validation. Most models are trained and tested on pre-selected datasets without evaluation on independent cohorts, which may fail to account for real-world variations such as motion artifacts and low-contrast imaging. Some studies have reported exceptionally high DSC values, which may reflect potential issues such as data leakage, non-independent testing, or overfitting, particularly in small or homogeneous datasets. In addition, integrating such segmentation tools into clinical practice remains challenging, as they are often implemented as standalone add-on modules rather than being embedded within existing workflows. This challenge is further compounded by the fact that many clinical centers may lack the high-performance GPU infrastructure required for real-time neural network inference.

Looking ahead, future research should prioritize both the generalizability and clinical applicability of ILT segmentation. A critical step in this direction is the development of large, publicly accessible datasets with standardized annotations, which would significantly improve model robustness and external validity. Collaborative research groups, such as VASCUNET, the European Research Hub (ERH), or the European Vascular Research Collaborative (EVRC), are actively developing large-scale datasets based on real-world vascular surgery data [77]. The Vascular-AID consortium is also collecting data from AAA patients across multiple European centers, aiming to collect multimodal data including clinical records, radiological imaging, proteomics, and genomics from 5000 patients with AAA [78].

Achieving this objective would require coordinated efforts among clinical centers and research institutions. In parallel, time-efficient annotation strategies, such as semi-supervised learning or active learning methods, offer promising solutions to alleviate the annotation burdens while maintaining acceptable ground truth quality across multiple datasets. These pragmatic solutions may offer a viable pathway to scaling dataset size and diversity in the near term, while longer-term efforts toward comprehensive repositories continue to mature. From a modeling perspective, techniques like domain adaptation and multi-task learning represent important directions. Domain adaptation enables models to perform robustly across heterogeneous data sources with minimal retraining, while multi-task frameworks could simultaneously perform segmentation and deliver clinically actionable outputs, such as diameter estimation or rupture risk prediction. Additionally, incorporating longitudinal CTA and modeling temporal changes in aneurysm morphology may support more accurate surveillance and risk stratification.

Although PubMed and Web of Science were selected for their broad interdisciplinary coverage, we acknowledge that not searching specialized technical databases such as IEEE Xplore or the ACM Digital Library may have excluded certain relevant computer science studies. Preliminary checks indicated that many key IEEE-published studies were already indexed in the Web of Science results, supporting our database selection strategy. In addition, although all titles, abstracts, and full texts were independently screened by two reviewers, we acknowledge that formal inter-reviewer agreement statistics and standardized quality-assessment tools were not applied. Discrepancies in study selection were resolved through structured discussion and consensus, and substantial overlap between reviewers’ choices helped ensure consistency. Methodological rigor was maintained through predefined eligibility criteria and a consensus decision process.

Overall, the integration of DL ILT segmentation into AAA analysis, particularly for ILT, holds significant promise for advancing preoperative planning and postoperative surveillance. However, meaningful clinical translation will require more than algorithmic innovation alone. Key challenges remain in enhancing data accessibility, improving model generalizability and ensuring seamless integration into clinical workflows. Ultimately, bridging the gap between research progress and clinical application in AAA segmentation will require a multi-dimensional effort that integrates technical excellence with practical clinical requirements and understanding.

## 5. Conclusions

This review provides a structured overview of DL segmentation methods for AAA, with a particular focus on ILT. We highlight recent advances in 2D, 3D, and hybrid models, as well as their clinical applications in preoperative planning and postoperative monitoring. Multiview fusion models consistently outperformed other 2D approaches, while in 3D segmentation, the original 3D U-Net remains a robust and widely used baseline. While an increasing number of DL segmentation models have demonstrated strong performance in segmenting ILT, relatively few approaches have successfully achieved accurate and automated delineation of all major AAA components. Nevertheless, those comprehensive approaches often report superior segmentation fidelity and enhanced clinical utility.

The core contribution of this review lies in consolidating key advances, critically evaluating current methodological gaps, and highlighting emerging solutions. These approaches represent promising directions for improving scalability, robustness, and clinical relevance in future AAA segmentation research.

## Figures and Tables

**Table 1:** Summary of studies that used a DL approach for intraluminal thrombus (ILT) segmentation on CT/CTA images from patients with abdominal aortic aneurysm (from 2016 to 2025).

Reference	Dataset and Sample Size	Segmentation Target	Model/Method	Performance Measures
López-Linares et al., 2017 [28]	13 postoperative contrast-enhanced CTAs	ILT	Fully Convolutional Networks and a Holistically Nested Edge Detection Network 2D	ILT DSC = 0.89
Wang et al., 2018 [29]	22 contrast-enhanced CTAs and 22 MRIs of the same patients with AAA	For CT, aorta wall, lumen, ILT, and calcium deposits; for MRI, aorta wall, lumen, and ILT	Fusion-based Deep Convolutional Neural Network 2D	Mean ACC = 0.988
López-Linares et al., 2017 [30]	20 postoperative and 18 preoperative contrast-enhanced CTAs	ILT only	Fully Convolutional Networks and a Holistically Nested Edge Detection Network 2D	Postoperative ILT DSC = 0.855 ± 0.065; Preoperative ILT DSC = 0.697 ± 0.132
López-Linares et al., 2019 [31]	28 postoperative CTAs of patients with an infrarenal AAA and who have been treated with EVAR	ILT only	Synthetic Shape Model + 2D DCNN 2D	ILT DSC = 0.84 ± 0.01
Caradu et al., 2021 [32]	100 contrast-enhanced CTA scans of patients with infrarenal AAA treated by EVAR, including both pre- and post-EVAR multidetector follow-up scans	Lumen and ILT	PRAEVAorta for segmentation: image preprocessing and segmentation of the aortic lumen and thrombus 2D	ILT DSC = 0.81 ± 0.10
Lareyre et al., 2021 [33]	40 contrast-enhanced and 53 lower-contrast CTAs of infrarenal AAA patients undergoing elective surgery from multi data centers	Lumen, spine, and ILT	Fully CNN with a U-Net architecture 2D	ILT DSC = 0.89
Brutti et al., 2022 [34]	85 contrast-enhanced CTAs from multi-data centers	ILT and lumen	Multi-view integration approach, 3D + 2D × 3 views 2D + 3D	DSC = 0.89 ± 0.04
Jung et al., 2022 [35]	60 postoperative CTAs of patients with AAA	ILT only	2D Bi-CLSTM-based thrombus ROI segmentation method combined with Mask R-CNN 2D	ILT DSC = 0.89
Abdolmanafi et al., 2022 [36]	6030 CT slices from abdominal CT of 56 patients	Lumen, thrombus, and calcification	2D multi-stage DL pipeline employing a 2D Residual U-Net with dilated convolutions 2D	Calcified ILT ACC = 0.91 Non-calcified ILT ACC = 0.85
Hwang et al., 2022 [37]	Thrombus CTA scan images from 60 unique patients	ILT only	ResNet50 as backbone combined with a feature pyramid network 2D	ILT F1 = 0.9197
Caradu et al., 2022 [38]	101 CT scans within 48 postoperative CTs	Lumen and ILT	PRAEVAorta 2D	ILT DSC = 0.848 ± 0.100, JAC = 0.747 ± 0.133
Wang et al., 2022 [39]	340 contrast-enhanced CTs of patients of infer-renal AAA with ILT from a single center	Lumen and ILT	DeepLabv3+-based DCNN model with ResNet-50 backbone 2D	ILT IOU = 0.8650 ± 0.0033 Mean IOU = 0.9078 ± 0.0029
Wang et al., 2022 [40]	340 contrast-enhanced CTs of patients of infer-renal AAA with ILT from a single center	Lumen and ILT	DeepLabv3+-based DCNN model with ResNet-50 backbone 2D	ILT IOU = 0.8650 ± 0.0033 Mean IOU = 0.9078 ± 0.0029
Kongrat et al., 2022 [41]	60 CTAs from a single center: 8 of normal subjects 14 of AAA patients 38 of AAA patients with thrombus	Lumen and ILT	3D U-Net	ILT DSC = 0.9868
Chandrashekar et al., 2022 [42]	75 preoperative CTAs (284,624 CTA axial slices and 145,320 NCCT axial slices)	Aorta, lumen, and wall structure/ILT	2D Attention-based U-Net	ILT DSC = 87.2 ± 6.3%
Chandrashekar et al., 2023 [43]	75 patients with paired NCCTs and CTAs (11243 pairs of images); 200 independent cases with paired NCCTs and CTAs (29,468 pairs of images)	Lumen and ILT	2D Attention-based U-Net	ACC = 0.935
Mu et al., 2023 [44]	70 CTA scans	Lumen and ILT	Context-aware cascaded U-Net integrated with a Residual 3D U-Net with an auto-context 3D U-Net structure using an auto-context mechanism 3D	ILT DSC = 0.804 Lumen DSC = 0.945
Spinella et al., 2023 [45]	73 thoraco-abdominal CTAs (48 AAAs, 25 healthy controls) from 11 scanners	Lumen and ILT	A pipeline including a U-Net structed localization and a 2.5D CNN combined with a multi-view integration approach 2D	ILT DSC = 0.93
Kim et al., 2024 [46]	Prospective cohort of patients with AAA (Oxford Abdominal Aortic Aneurysm Study) - 94 patients at 12 months - 79 patients at 24 months	Lumen, ILT, and calcification,	nnUnet, UNETR, and SwinUNETR tested 3D	2D–3D U-Net ILT DSC = 0.782 ± 0.170 and HD95 = 11.616 ± 13.021mm
Lyu et al., 2024 [47]	80 CTAs of AAA patients	ILT	Mixed-scale-driven Multiview perception network (M2Net) model 3D	ILT DSC = 0.884 ± 0.022, HD95 = 1.172 ± 0.493, IOU = 0.797 ± 0.034
Robbi et al., 2025 [48]	Public dataset [49,50] Pipeline validation: 20 pre-CTAs from multiple datasets [49,51,52,53]	Lumen, ILT, calcification, and abdominal branches	BRAVE (Blood Vessels Recognition and Aneurysms Visualization Enhancement) including nnUnet for initial segmentation and SegResNet for the refinements 3D	AAA sac ILT DSC = 0.97 ± 0.03, HD = 4.52 ± 5.02
Zhang et al., 2025 [54]	63 CTAs of AAA patients from two scanners	Lumen and ILT	SDLU-Net 3D	ILT DSC = 0.828, HD95 = 17.330; Lumen DSC = 0.924, HD95 = 8.024

ILT: Intraluminal thrombus; EVAR: endovascular aneurysm repair; CNN: convolutional neural network; SDLU: similarity-based dynamic linking; DSC: Dice similarity score; HD95: Hausdorff distance 95%; IOU: intersection over union; ACC: accuracy; CT: computed tomography; CTA: computed tomography angiography.

**Table 2:** Summary of public datasets reviewed in this study.

Dataset Name	Number of Cases	Imaging Modality	Annotation Rule	ILT Annotation Available
AVT [52]	56 volumes	CTA	Aorta only	No
Vascular Model Repository [51]	91 volumes about aorta	CT/CTA/MRI/Ultrasound	Aorta only	No
TotalSegmentator [49]	1204 volumes	CT	104 anatomical structures (27 organs, 59 bones, 10 muscles, 8 vessels)	No
AortaSeg24 [66]	100 volumes	CTA	23 aortic branches and Society for Vascular Surgery/Society of Thoracic Surgeons zones	No

## Data Availability

Not applicable. No new data were created or analyzed in this study.

## Supplementary Materials

The following supporting information can be downloaded at: https://www.mdpi.com/article/10.3390/jcm14238497/s1, Table S1: Literature search terms; Table S2: Checklist for eligibility assessment of included studies; Table S3: Summary of different segmentation performance metrics.

## Abbreviations

The following abbreviations are used in this manuscript:

AAA	Abdominal aortic aneurysm
AI	Artificial intelligence
AL	Active learning
Bi-CLSTM	Bi-directional convolutional long short-term memory
CT	Computed tomography
CTA	Computed tomography angiography
CNN	Convolutional neural network
DL	Deep learning
DSC	Dice similarity coefficient
DUS	Duplex ultrasound
EVAR	Endovascular aneurysm repair
FCN	Fully convolutional network
HD95	Hausdorff distance 95%
ILT	Intraluminal thrombus
IOU	Intersection over union
NCCT	Non-contrast computed tomography
OAR	Open aneurysm repair
SDLU	Similarity-based dynamic linking
WSS	Wall shear stress
