# Supplementary material for: Automatic Segmentation of Intraluminal Thrombus in Abdominal Aortic Aneurysms Based on CT Images: A Comprehensive Review of Deep Learning-Based Methods
Source: J Clin Med. 2025 Nov 30;14(23):8497. doi: 10.3390/jcm14238497 (PMC12692895; doi:10.3390/jcm14238497)
Supplement: Supplementary file 1 [file jcm-14-08497-s001.zip › jcm-3895235-supplementary.pdf]

Supplementary

Table S1: Literature search terms.

<table>
<tr><td>Artificial intelligence</td><td rowspan="7">AND</td><td rowspan="2">Segmentation</td><td rowspan="7">AND</td><td>Intraluminal thrombus</td></tr>
<tr><td>OR</td><td>OR</td></tr>
<tr><td rowspan="3">Deep learning</td><td rowspan="3">OR</td><td>Aneurysm</td></tr>
<tr><td>OR</td></tr>
<tr><td>Aortic aneurysm</td></tr>
<tr><td>OR</td><td rowspan="2">Measurement</td><td>OR</td></tr>
<tr><td>Neural network</td><td>Abdominal aortic aneurysm</td></tr>
</table>

Table S2: Checklist for eligibility assessment of included studies.

**No.**	**Check list**
1	Was this study published within the predefined time interval?
2	Is the imaging modality consistent with the inclusion criteria (CTA or non-enhanced CT)?
3	Does the study focus on the region of AAA?
4	Does the study address the medical image segmentation task?
5	Does the study achieve individual intraluminal thrombus segmentation?
6	Does the study use deep learning methods?
7	Is sufficient information provided about the model architecture and training details?
8	Are performance metrics reported (DSC, IoU or F1)?

Table S3: Summary of different segmentation performance metrics

Measurements metric	Explanations
Dice similarity score (DSC)	Compare the overlap between two segmentations. $$DSC = \frac{2\|A \cap B\|}{A \cup B}$$
Intersection over union (IOU) / Jaccard index	Compare the overlap between two segmentations. $$IOU = \frac{\|A \cap B\|}{\|A \cup B\|}$$
Hausdorff distance 95% (HD95)	The HD presents longest distance of how far two subsets of a metric space are from each other. $$HD(A,B) = max\{\sup_{a\in A} d(a,B), \sup_{b\in B} d(A,b)\}$$ $$d(a,B) = \min_{b\in B} d(a,b)$$ By using the 95th percentile, HD95 reduces the influence of extreme outliers and provides a more robust estimate of the typical boundary mismatch.
Precision	The fraction of relevant instances among the retrieved instances. $$Precision = \frac{TP}{TP + FP}$$
Recall	The fraction of relevant instances that were retrieved. $$Recall = \frac{TP}{TP + FN}$$
F-score(F1)	A measure of predictive performance, which is the harmonic mean of precision and recall and resulting a same value of DSC. $$F1 = \frac{2}{recall^{-1} + precision^{-1}} = \frac{2 \times Precision \times Recall}{Precision + Recall} = \frac{2TP}{2TP + FP + FN}$$
Accuracy (ACC)	A statistical measure of how well a binary classification test correctly identifies or excludes a condition. $$ACC = \frac{TP + TN}{TP + TN + FP + FN}$$
Volume similarity	Compare how close the volumes of two segments are. $$VS = 1 - \frac{\|V_{seg} - V_{gt}\|}{V_{seg} + V_{gt}}$$
